# Dataset on the patterns of electricity consumption in public universities in southwestern Nigeria

**DOI:** 10.1016/j.dib.2018.09.056

**Published:** 2018-09-26

**Authors:** Sunday Segbenu Nunayon

**Affiliations:** Department of Architecture and Civil Engineering, City University of Hong Kong, Tat Chee Avenue, Kowloon, Hong Kong, China

**Keywords:** Electrical energy consumption, Energy efficiency, Electricity consumption pattern, Energy management, Public universities, Nigeria

## Abstract

In this data article, a thorough dataset on patterns of electricity use in Nigerian public universities was presented. Three relatively old public universities with staff and students’ halls of residences were purposively selected. The selected universities were Obafemi Awolowo University, Ile-Ife (OAU), Federal University of Technology, Akure (FUTA), and University of Ibadan (UI). Data were obtained through the administration of a structured questionnaire on electricity end users in the universities sampled. For the data collection, the electricity end users in public universities were stratified into users in university staff offices, staff residences, student residences, and commercial units. Electricity users were selected using systematic random sampling and accidental sampling techniques. In OAU, FUTA and UI, 217, 137 and 164 students, respectively were sampled; 30, 3, and 61 households in OAU, FUTA, and UI, respectively were sampled while 28, 6, and 18 commercial units in OAU, FUTA and UI, respectively were also sampled. The sample size determined for staff in OAU, FUTA and UI were 139, 81 and 182, respectively. The data obtained were analyzed using radar charts. The information provided in this data article will encourage investigation into electricity management strategies, critical success factors for electricity management, planning, and policy formulation towards the realization of sustainable campuses.

**Specification table**

**Table:** 

Subject area	Building
More specific subject area	Electricity consumption in buildings
Type of data	Figures
How data was acquired	Administration of questionnaire intended to collect the respondents’ electricity consumption data, which included device and operational time (kilowatt per hour).
Data format	Raw and analyzed
Experimental factors	The electricity consumption formula shown below was used to determine the electricity consumption level:
	(1) $$Q=\sum_{j=1}^{n}C_{k}\cdot H_{j}$$
	$\begin{array}{c} Where\;:\\ Q=\mathrm{Energy}\;\mathrm{consumption}\;\mathrm{of}\;\mathrm{equipment};\\ C_{k}=\mathrm{Capacity}\;\mathrm{of}\;\mathrm{equipment}\;k;\\ H_{j}=\mathrm{Hour}\;\mathrm{of}\;\mathrm{use}\;\mathrm{per}\;\mathrm{day};\;\mathrm{and}\\ n=\mathrm{Number}\;\mathrm{of}\;\mathrm{user}. \end{array}$
Experimental features	The electricity consumption data were analyzed to show the pattern of electricity use in public universities in Southwestern Nigeria.
Data source location	The electricity use data presented in this article were obtained at OAU, FUTA, and UI, in Southwestern Nigeria
Data accessibility	Data on electricity use patterns are provided with this article
Related research articles	i. K, Steemers, G.Y. Yun, Household energy consumption: a study of the role of occupants, *Building Research & Information*, 37(5-6) (2009) 625–637. ii. M. Link, L. Sela, *Analysis of University Water and Energy Consumption to Support Management and Conservation Strategies*, World Environmental and Water Resources Congress, (2018) 152–161. iii. T. Lhendup, S. Lhundup, T. Wangchuk, Domestic energy consumption patterns in urban Bhutan. *Energy for Sustainable Development*, 14(2) (2010) 134–142.

**Value of the data**

• The data will foster more empirical investigation for a better understanding of areas of improvement in electricity use [1].
• The data provided in this article is useful for the development of electricity use models, energy audit, and energy management practices [2], [3].
• The data provided will assist the government and the management of public universities in result-oriented planning, budgeting, and decision-making [4].
• The provision of this data will propel the achievement of the sustainable campus environment [5], [6], [7], [8].

## 1 Data

**Fig. 1:**
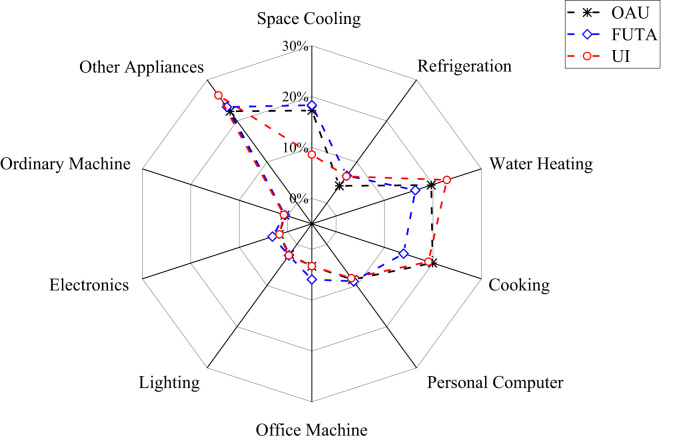
Electricity end-use in Nigerian public universities.

**Fig. 2:**
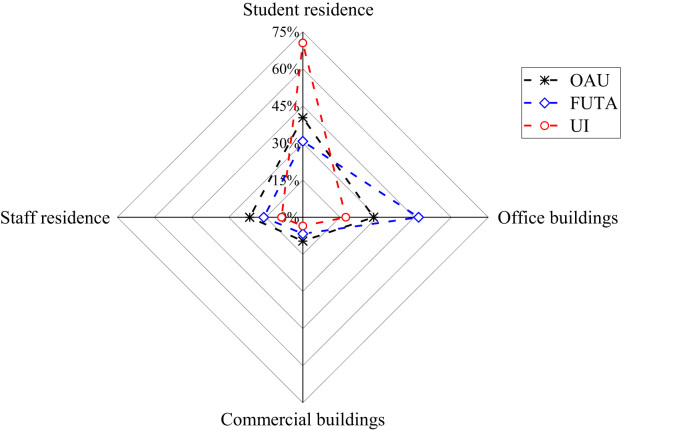
Electricity end-use by stratification.

**Fig. 3:**
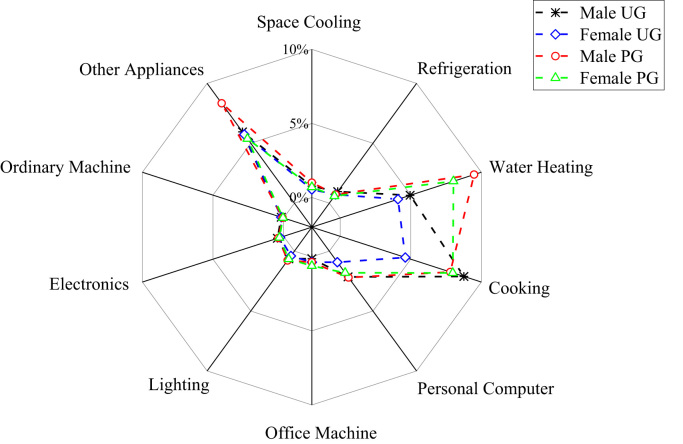
Gender disparity of electricity end-use among undergraduate and postgraduate students in OAU.

**Fig. 4:**
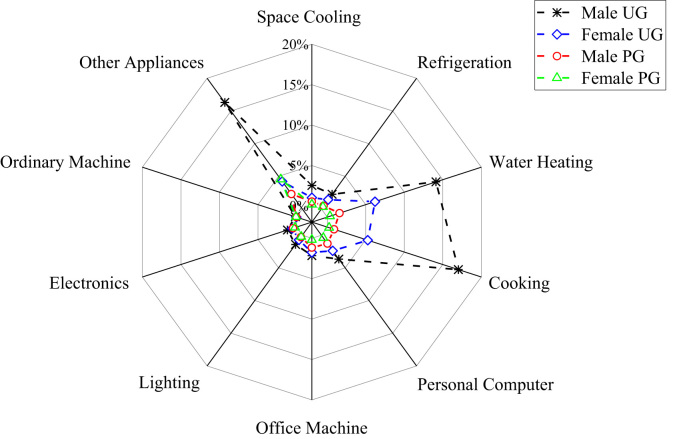
Gender disparity of electricity end-use among undergraduate and postgraduate students in FUTA.

**Fig. 5:**
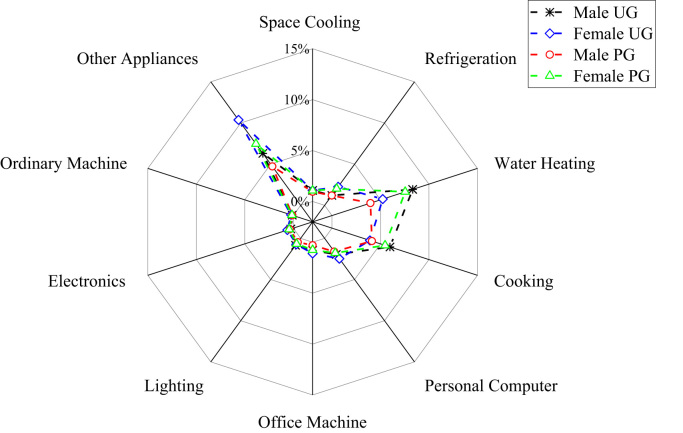
Gender disparity of electricity end-use among undergraduate and postgraduate students in UI.

**Fig. 6:**
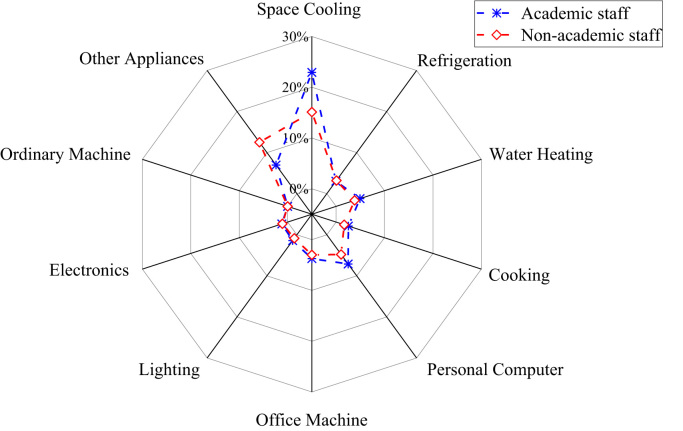
Comparison of electricity end-use among university staff in OAU.

**Fig. 7:**
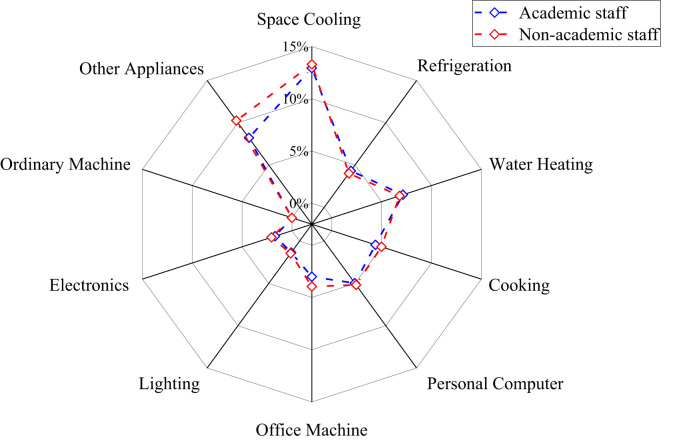
Comparison of electricity end-use among university staff in FUTA.

**Fig. 8:**
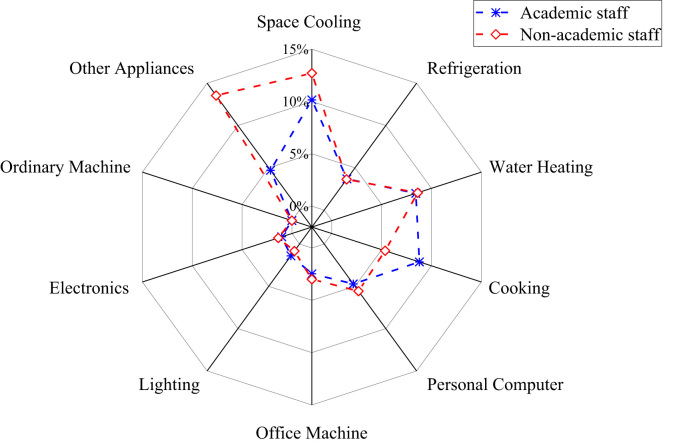
Comparison of electricity end-use among university staff in UI.

The patterns of electricity consumption were demonstrated from different perspectives (Fig. 1, Fig. 2, Fig. 3, Fig. 4, Fig. 5, Fig. 6, Fig. 7, Fig. 8). Fig. 1 shows the share of electricity consumption by different end-uses, ranging from 0.54% to 22.32% in OAU, 0.57% to 23.38% in FUTA and 0.76% to 26.21% in UI [9], [10]. Fig. 2 indicates that the respective percentage electricity consumption of students’ residences, office buildings, commercial units and staff residences were 40.25%, 28.66%, 9.61% and 21.48% in OAU, 30.70%, 40.81%, 6.70% and 15.79% in FUTA, and 70.50%, 17.39%, 3.52% and 8.59% in UI [11]. The radar plots showing comparisons between electricity use in male and female residences for both undergraduate (UG) and postgraduate (PG) students are illustrated in Fig. 3, Fig. 4, Fig. 5, accounting for OAU, FUTA and UI, respectively. Male hostels for both UG and PG students in OAU and FUTA consume more electricity than female hostels. The proportions of the total student consumption observed, respectively, for male UG, female UG, male PG, and female PG include 24.93%, 17.82%, 31.14%, 26.11% in OAU and 60.76%, 23.75%, 8.97%, 6.72% in FUTA. However, in UI, female hostels were found to consume more electricity than male hostels in the following order: 26.86%, 28.01%, 17.79% and 27.34% for male UG, female UG, male PG, and female PG, respectively. Fig. 6, Fig. 7, Fig. 8 show comparisons between the electricity consumption of academic and non-academic staff offices. The consumption quota for the academic and non-academic staff offices, respectively were 53.90% and 46.10% in OAU, 48.02% and 51.98% in FUTA, 45.38% and 54.62% in UI [12], [13], [14], [15], [16].

## 2 Experimental design, materials, and methods

The quantitative technique was adopted. The instrument for data collection was a structured questionnaire. A multi-stage sampling technique was used for the data collection [17]. In the first stage, purposive sampling was used to select relatively old public universities with staff and students’ halls of residences. In this regard, Obafemi Awolowo University, Ile-Ife (OAU); Federal University of Technology, Akure (FUTA); and University of Ibadan (UI) were selected. The second stage was the stratification of electricity users into staff offices, staff residences, student residences, and commercial units. Student halls were categorized into two: undergraduate male, undergraduate female, and postgraduate male and postgraduate female hostels. Student hostels were purposively selected to capture variation in gender and levels of study. In OAU, FUTA and UI, Moremi, Jadesola Akande and Awolowo halls, respectively were selected as representatives of female undergraduate hostels in the universities, while Awolowo, Peter Adeniyi, and Independence halls were selected for male undergraduate students in OAU, FUTA, and UI respectively. Murtala Muhammed Postgraduate hall in OAU, FUTA Postgraduate hall in FUTA and Abdusalam Abubakar Postgraduate hall in UI for both Male and Female students were also sampled.

The populations of students occupying these hostels earlier determined were 1228, 1200 and 1618 for Moremi, Jadesola Akande and Awolowo halls, respectively; 2,032, 1142 and 956 for Awolowo, Peter Adeniyi and Independence halls, respectively, while there were 1072 (408 female, 664 male), 400 (120 female, 280 male) and 700 (420 female, 280 male) in Murtala Muhammed, FUTA and Abdusalam Abubakar Postgraduate halls, respectively. One out of every twenty (5%) students were selected in each hall. In all, 217, 137 and 164 students were sampled in OAU, FUTA, and UI, respectively. The preliminary investigation also showed that in OAU, FUTA, and UI, there were 600, 50 and 1212 households, respectively in staff residential quarters while there were 552, 115 and 350 shops, respectively to constitute the sample frame for both staff housing units and business units. One out of every twenty (5%) households and shops were selected. Using this method, 30, 3 and 61 households in OAU, FUTA, and UI, respectively were sampled while 28, 6 and 18 business units in OAU, FUTA, and UI, respectively were also sampled. For staff offices, accidental sampling was used across academic, administrative and other cadres of staff in the three universities. The sample size determined for staff in OAU, FUTA and UI were 139, 81 and 182, respectively.

The amount of electricity consumption was calculated from Eq. (1). For the sake of clarity and potential transmission, the statistical analyses of the electricity data have been presented using radar charts. Suitable deductions and discussions based on these plots will give a valuable understanding that is required for valid conclusions.
