# Supplementary material for: Dataset on the patterns of electricity consumption in public universities in southwestern Nigeria
Source: Data Brief. 2018 Sep 26;21:1–7. doi: 10.1016/j.dib.2018.09.056 (PMC6176851; doi:10.1016/j.dib.2018.09.056)
Supplement: Supplementary file 1 — Transparency document [file mmc1.pdf]

CONFLICT OF INTEREST FORM

I wish to confirm that there are no known conflicts of interest associated with this publication. I confirm that there are no other persons who satisfied the criteria for authorship but were not listed.

Signed

Sunday Segbenu Nunayon (16/08/2018)
